# Media multitasking, mind-wandering, and distractibility: A large-scale study

**DOI:** 10.3758/s13414-019-01842-0

**Published:** 2019-08-07

**Authors:** Wisnu Wiradhany, Marieke K. van Vugt, Mark R. Nieuwenstein

**Affiliations:** 1 Department of Experimental Psychology, University of Groningen, Grote Kruistraat 2/1, 9712 TS Groningen, the Netherlands; 2 Bernoulli Institute of Mathematics, Computer Science and Artificial Intelligence, University of Groningen, Groningen, the Netherlands

**Keywords:** Media multitasking, Cognitive control, Working memory, Change detection, Mind-wandering

## Abstract

Previous studies suggest that frequent media multitasking – the simultaneous use of different media at the same time – may be associated with increased susceptibility to internal and external sources of distraction. At the same time, other studies found no evidence for such associations. In the current study, we report the results of a large-scale study (N=261) in which we measured media multitasking with a short media-use questionnaire and measured distraction with a change-detection task that included different numbers of distractors. To determine whether internally generated distraction affected performance, we deployed experience-sampling probes during the change-detection task. The results showed that participants with higher media multitasking scores did not perform worse as distractor set size increased, they did not perform worse in general, and their responses on the experience-sampling probes made clear that they also did not experience more lapses of attention during the task. Critically, these results were robust across different methods of analysis (i.e., Linear Mixed Modeling, Bayes factors, and extreme-groups comparison). At the same time, our use of the short version of the media-use questionnaire might limit the generalizability of our findings. In light of our results, we suggest that future studies should ensure an adequate level of statistical power and implement a more precise measure for media multitasking.

**Electronic supplementary material:**

The online version of this article (10.3758/s13414-019-01842-0) contains supplementary material, which is available to authorized users.

## Introduction

Media multitasking, the act of consuming multiple media streams simultaneously, has become increasingly prevalent, with a recent report indicating that US adolescents consumed 10.5 h of media content in 7.5 h per day by multitasking (Rideout, Foehr, & Roberts, 2010). In light of this development, researchers have begun to examine how the frequency of media multitasking relates to various indices of personality, mental health, and cognition (for reviews, see Carrier, Rosen, Cheever, & Lim, 2015; Courage, Bakhtiar, Fitzpatrick, Kenny, & Brandeau, 2015; Uncapher et al., 2017; Van Der Schuur, Baumgartner, Sumter, & Valkenburg, 2015). On the one hand, several studies showed evidence for a weak association of media multitasking with self-report measures of impulsivity and sensation-seeking (e.g., Minear, Brasher, McCurdy, Lewis, & Younggren, 2013; Sanbonmatsu, Strayer, Medeiros-Ward, & Watson, 2013) and attention-deficit hyperactivity disorder (ADHD)-related symptoms (Baumgartner, van der Schuur, Lemmens, & te Poel, 2017a; Magen, 2017; Uncapher, Thieu, & Wagner, 2016). On the other hand, however, studies exploring the correlates of media multitasking in laboratory measures of selective attention, working memory, and executive control have thus far produced less compelling results. Specifically, while some studies in this domain suggest that media multitasking might be associated with increased vulnerability to distractors (e.g., Ophir, Nass, & Wagner, 2009), others suggest that habitual media multitaskers may perform worse across various cognitive tasks, regardless of the presence of distractors (e.g., Uncapher, Thieu, & Wagner, 2016). These sets of finding suggest two possible mechanisms by which media multitasking could affect cognitive task performance, namely that media multitaskers are affected by the presence of external distraction or, alternatively, that they might get distracted by something else, unrelated to the task. In the next two sections, we will evaluate the evidence of these in further detail.

### The external distraction hypothesis

The first subset of studies suggests that people who frequently engage in media multitasking behavior may have problems in filtering out distracting information from their immediate environment. We refer to this as the external distraction hypothesis.

#### Evidence for the external distraction hypothesis

To start, Ophir et al. (2009) showed that heavy, compared to light, media multitaskers (HMMs and LMMs, respectively) performed worse in a change-detection task with varying numbers of distractors. Specifically, in this study, participants had to memorize two target objects that could be shown together with zero, two, four, or six distractor objects. The results showed that HMMs, but not LMMs, performed worse as the number of distractor objects increased. In addition, HMMs responded slower in an AX-CPT task when the targets appeared amongst distractors, but not when the targets were shown without distractors, thereby suggesting that media multitasking may be associated with increased susceptibility to distraction from task-irrelevant stimuli in the environment. Further supporting this idea, Moisala et al. (2016) found that HMMs made more mistakes than LMMs when they were instructed to attend to stimuli in one modality (e.g., visual) while ignoring stimuli from another modality (e.g., auditory).

One possible explanation for these previously observed associations between media multitasking and task performance is that HMMs experience increased susceptibility to distraction due to the development of a breadth-biased cognitive control style (Lin, 2009). Specifically, since the media environment is saturated with information and one piece of seemingly irrelevant information may be valuable later, HMMs might develop the tendency to distribute their focus of attention more equally across multiple streams of information. As a consequence, they might become less sensitive in distinguishing relevant from irrelevant pieces of information. Indeed, supporting this idea, HMMs were reported to be better in a sensory-integration task in which a task-irrelevant auditory stimulus could help guide attention towards a target in a dynamic visual-search task if the tone was presented simultaneously with the blinking of the target in the search display (Lui & Wong, 2012; see also Van der Burg, Olivers, Bronkhorst, & Theeuwes, 2008). In other words, this study could be interpreted to suggest that a breadth-biased focus of attention caused the HMMs to be more sensitive to the task-irrelevant information that was in this case beneficial for task performance.

Another possible explanation for increased distractibility in HMMs is that HMMs have a reduced ability to exert top-down control over attentional selection (Cain & Mitroff, 2011). This account derives from the results of a visual search task in which participants had to respond to a target that appeared within one of several shapes that were all shown in the same color. On some trials, a shape with an oddball color was present, and the researchers examined whether HMMs and LMMs differed in their ability to ignore this oddball distractor depending on the likelihood that this oddball could contain the target. Specifically, in the *never* block, participants were validly instructed that the target would never appear in the oddball distractor color while in the *sometimes* block, the target could appear in the the oddball color on some of the trials. The results showed that LMMs were less affected by the presence of the oddball distractor in the *never* block than in the *sometimes* block, indicating that they used the instruction to modulate their visual attention to filter out the oddball distractor while HMMs showed comparable response times (RTs) in the *never* and *sometimes* blocks, indicating that they did not use the instructions to modulate their attention. Taken together, these findings suggest that media multitasking may be associated with increased susceptibility to distraction from task-irrelevant stimuli in the environment, and this may arise from a breadth-biased focus of attention and/or a reduced ability to exert top-down control over attentional selection.

#### Evidence against the external distraction hypothesis

While studies have suggested multiple lines of evidence in favor of the external distraction hypothesis, evidence against the hypothesis has also been accumulating. Specifically, the external distraction hypothesis appears to be at odds with the fact that various studies did not find that HMMs perform worse in the presence of distractors, for example in a change-detection task (Cardoso-Leite et al., 2015; Gorman & Green, 2016; Uncapher et al., 2016; Wiradhany & Nieuwenstein, 2017) and in an AX-CPT task (Cardoso-Leite et al., 2015). Moreover, our recent meta-analysis (Wiradhany & Nieuwenstein, 2017) showed that out of 39 tests of the external distraction hypothesis, only ten showed significantly stronger distractibility in HMMs, whereas three showed significantly stronger distractibility in LMMs, and the remaining 26 showed no significant difference. The pooled effect size for the association between media multitasking and external distractibility was weak (*Cohen’s d =* .17), and this association turned non-significant after we corrected for the presence of small-study bias.

### The internal distraction hypothesis

A second hypothesis about the relationship between media multitasking and performance on cognitive tasks proposes that media multitasking is associated with worse task performance overall, and this might be due to participants being distracted by something unrelated to the task (e.g., Uncapher et al., 2016). We refer to this as the internal distraction hypothesis.

#### Evidence for the internal distraction hypothesis

In a change-detection task with two targets and varying numbers of distractors, Uncapher et al. (2016) found that heavy media multitasking was associated with worse performance regardless of the presence of distractors (see also Wiradhany & Nieuwenstein, Exp.1). This was true regardless of whether participants tried to detect changes in orientations of red and blue rectangles (Exp. 1 in Ophir et al., 2009) or line-drawings of everyday objects (their Exp. 2) and, importantly, regardless of whether only the extreme multitaskers (i.e., HMMs and LMMs) or all participants were considered in the analysis. Further, they found that HMMs were less able to discriminate previously presented target and distractor objects in the change-detection task from novel objects in a subsequent long-term memory-recognition test.

In interpreting these results, Uncapher et al. (2016) proposed that HMMs might experience “continual distraction by information not under experimental control” (p. 7), and further suggested that this might be due to a wider attentional scope during encoding and retrieval, thus resulting in lower performance. Here, taking insight from Uncapher et al.’s proposal that the distraction might not be under experimental control, we suggest that such continual distraction may be related to a difficulty in suppressing task-unrelated thoughts. Indeed, there has also been evidence to suggest that HMMs may experience mind-wandering – the presence of task-unrelated thoughts – more frequently, both in daily life (Ralph, Thomson, Cheyne, & Smilek, 2013) and while trying to memorize a video-recorded lecture (Loh, Tan, & Lim, 2016). These studies thereby offer support for the notion that HMMs might have difficulty in performing cognitive tasks due to problems in suppressing task-irrelevant thoughts.

This so-called internal distraction hypothesis may provide a possible account for other findings showing a general deficit of task performance in HMMs. This account may explain why HMMs perform worse in the Raven’s Progress Matrices (Minear et al., 2013); instead of deliberating sufficiently on the correct responses, they are distracted by task-unrelated thought and go with a less-deliberate response. Similarly, this hypothesis may provide an explanation for data showing that HMMs perform worse than LMMs in the OSPAN task (Sanbonmatsu et al., 2013), the count span task (Cain, Leonard, Gabrieli, & Finn, 2016), and the N-back task (Cain et al., 2016; Ophir et al., 2009; Ralph & Smilek, 2016) due to task-unrelated thought (see also Daamen, van Vugt, & Taatgen, 2016, for direct evidence of task-unrelated thinking during a complex working memory task).

#### Evidence against the internal distraction hypothesis

Although several studies have reported overall worse task performance of HMMs compared to LMMs, others have found that performance of HMMs and LMMs did not differ in tasks such as a change-detection task (Cardoso-Leite et al., 2015; Gorman & Green, 2016; Wiradhany & Nieuwenstein, 2017, Exp. 2), an N-back task (Edwards & Shin, 2017; Wiradhany & Nieuwenstein, 2017), a digit-span task (Baumgartner, Weeda, van der Heijden, & Huizinga, 2014), sustained attention tasks (Ralph, Thomson, Seli, Carriere, & Smilek, 2015), a task-switching paradigm (Alzahabi, Becker, & Hambrick, 2017; Baumgartner et al., 2014; Minear et al., 2013), an Eriksen flanker task (Murphy, McLauchlan, & Lee, 2017), and a Go/noGo task (Murphy et al., 2017; Ophir et al., 2009). In addition, one study found that HMMs performed better than LMMs. Specifically, in two experiments, Alzahabi and Becker (2013) found that HMMs performed better in a task-switching task. Lastly, some studies also failed to provide support for the idea that HMMs perform worse overall due to task-unrelated thoughts. Specifically, Ralph et al. (2015) reported that HMMs did not experience more frequent task-unrelated thought while performing a sustained-attention task. Collectively, these findings suggest that either the internal distraction hypothesis is incorrect, or that the internal distraction in HMMs only occurs during specific types of tasks.

### The current study

Taken together, it can be concluded that the results of previous studies on the association between media multitasking and performance on cognitive tasks are mixed. Some studies suggest that media multitasking is associated with increased susceptibility to distraction by task-irrelevant stimuli (i.e., the external distraction hypothesis), whereas others suggest that media multitasking is associated with worse performance overall, due to internally generated distraction (i.e., the internal distraction hypothesis), and yet others show no evidence for either of these associations.

In the current study, we collected data from a large sample of participants (N=261) to determine the respective roles of external and internal distraction in modulating task performance of media multitaskers. Participants completed a questionnaire for media multitasking and a visual change-detection task to assess their vulnerability to internal and external distraction. The change-detection task was similar to the task that was used in previous studies that provided evidence for the external (Ophir et al., 2009) and internal (Uncapher et al., 2016) distraction hypotheses. This task required participants to encode two target items (red rectangles) that could appear together with 0, 2, 4, or 6 distractor items (blue rectangles), thus enabling an assessment of the extent to which the presence of distractors interfered with memory for the target items (see also Vogel, McCollough, & Machizawa, 2005). Additionally, to assess whether HMMs and LMMs differed in terms of internal distraction, we first examined whether HMMs performed worse overall. Subsequently, if performance were worse overall, we would further examine whether this could be explained by an increase of task-unrelated thoughts during the experiment (see Smallwood & Schooler, 2015, for a review) by means of a mediation analysis (Fairchild & MacKinnon, 2009) to assess evidence for the internal distraction hypothesis.

We tested these hypotheses using linear mixed effects models that included the factors media multitasking, distractor set size, and mind-wandering across the entire sample of participants. Using linear mixed effects models has several advantages: It allows for analyzing a nested data structure and unbalanced design (Baayen, Davidson, & Bates, 2008; Bolker et al., 2009), which, as will become clear later, were present in our experiment. Additionally, compared to traditional ANOVAs, this method has also been proven to increase statistical power and lead to fewer false discoveries (Baayen et al., 2008; Bolker et al., 2009), and it allows for testing multiple covariates (Baayen et al., 2008; Yang, Zaitlen, Goddard, Visscher, & Price, 2014). Moreover, to examine whether the outcomes provided evidence against these hypotheses (i.e., whether there is evidence for the null hypothesis), we complemented the null-hypothesis significance test statistics with Bayes factors that can provide such evidence.

## Methods

### Participants

In total, 275 participants volunteered to take part in the study. Seven participants were excluded from data analysis because they did not complete the study, and another seven were excluded because they failed to respond in time to the task on more than 50% of trials (*M* = 89%, range 52.5–100%). The data from the remaining 261 participants were used for the statistical analysis. These 261 participants (159 female) had a mean age of 25.31 years (*SD* = 11.09). The study was approved by the Ethics Committee of the Psychology Department, University of Groningen. All participants provided informed consent prior to participating to the study.

### Materials and apparatus

The questionnaire to assess media multitasking and the change-detection task were implemented in OpenSesame 2.9.7 (Mathôt, Schreij, & Theeuwes, 2012). Data for 107 participants were collected in a lab equipped with ten computer set-ups that were shielded from view of each other. Data for the remaining 154 participants were collected in variable locations by second-year psychology students who could use their own computers and laptops to collect data, as part of an assignment for a research practicum course. These students were instructed to perform the experiment in a quiet, non-public location and to ask participants to turn off their mobile devices. The students who acted as experimenters stayed with participants during data collection to ensure that participants remained undisturbed and to provide opportunities to participants to ask questions if anything was unclear. Participants were debriefed after the data collection.

To test whether the results were different for data collected in the lab versus the data collected by students, we included the setting for data collection as a factor in our analyses. Our analysis showed that there were differences in demographics, media multitasking scores, and change-detection performance of the participants who were tested in the lab versus students using their own computers (see the Supplementary Materials of this article). Yet, these analyses also showed that the variance on the key variables in the two testing locations was equal and there was no difference in results pertaining to the relationship between media multitasking and performance on the change-detection task (see the Supplementary Materials, p. S1-S2). Together, these results indicate that while the data collected outside the lab may contain uncontrolled parameters, these parameters nevertheless do not seem to affect the variability in performance.

#### Media-Use Questionnaire

To measure media multitasking, we used the Short Media-Use Questionnaire (Baumgartner, Lemmens, Weeda, & Huizinga, 2017b). This questionnaire is a shortened version of a media-use questionnaire used in Baumgartner et al. (2014) and it is one of the many iterations of the Media-Use Questionnaire that was introduced in Ophir et al. (2009). All media-use questionnaires ask how often participants consume one type of media while consuming another at the same time across a range of different types of media, and then provide a composite metric of media multitasking, typically the Media Multitasking Index (MMI).

The scale that was introduced in Baumgartner et al. (2014) iterates the media pairing question over nine types of media: Print media, Television, Video on a computer, Music, Video/computer games, Phone calls, Instant/text messaging, Networking sites, and Other computer activities. The short version of this questionnaire, which was introduced in Baumgartner et al. (2017b), includes the nine most prevalent media pairs involving four types of media in a large sample of adolescents, namely TV, social network sites, instant messaging, and listening to music (see Baumgartner et al., 2017 for a description of the items). The response options are “never,” “sometimes,” “often,” and “almost always,” and these responses are assigned a score of 1, 2, 3, or 4, respectively. These responses are averaged, creating the Media Multitasking-Short (MMS) index. Importantly, in validating the short questionnaire, Baumgartner et al. (2017b) found that the variance captured in the short questionnaire explained a significant amount of variance of the long version of the questionnaire they used in 2014, and this was true regardless of whether they calculated the MMS; *r*(523) = .82 or the MMI using the formula provided in Ophir et al. (2009); *r*(523) = .84. Our motivation to use the MMS was supported further by the facts that participants could finish the short version of the questionnaire quickly (Baumgartner et al., 2017b) and that the MMS probes a more up-to-date set of media than the original questionnaire introduced by Ophir et al., which did not include Social Media.

#### Change-detection task

**Fig. 1:**
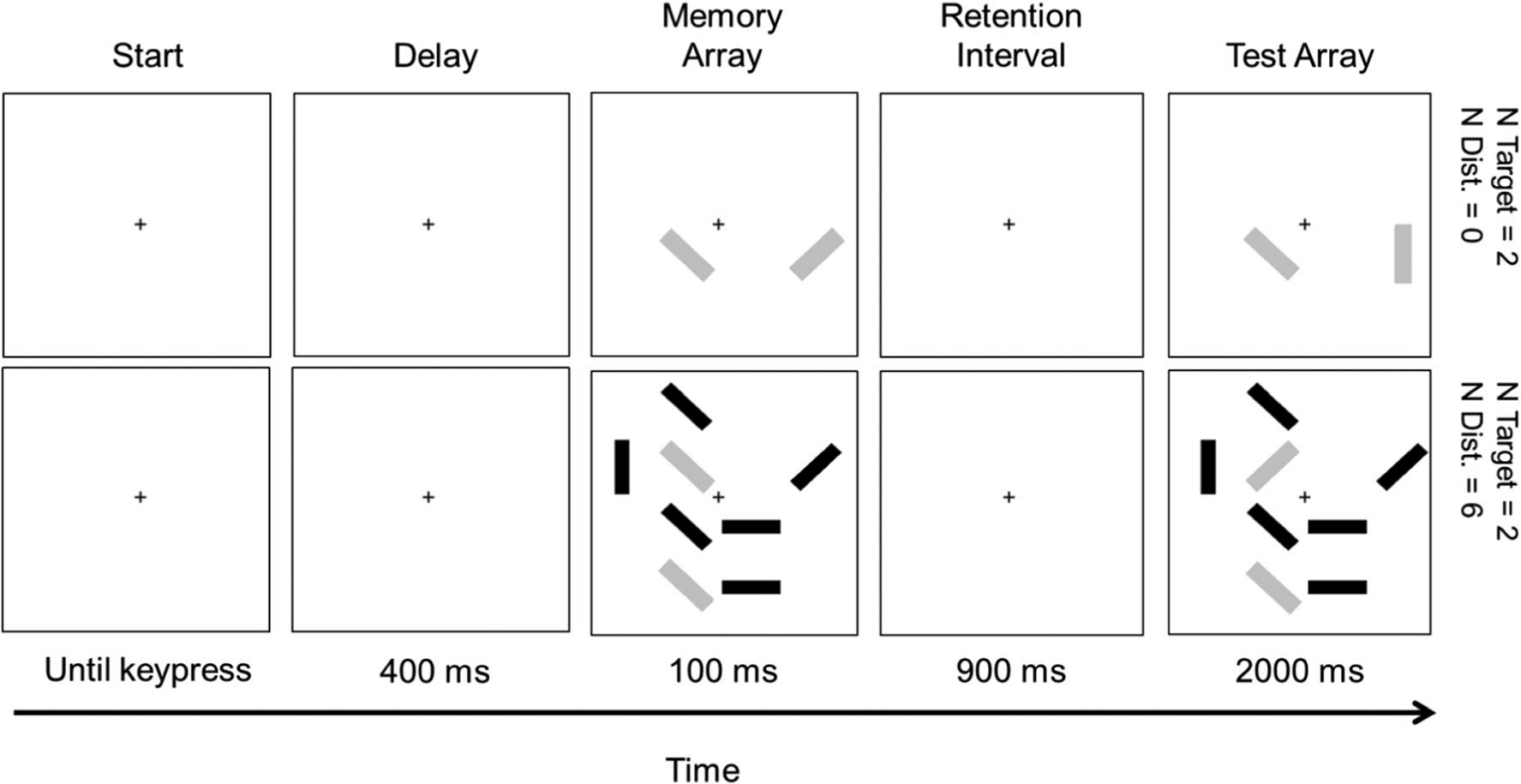
Two example trials from the change detection task, with zero and six distractors (upper and lower panels, respectively). Participants had to remember the orientations of two red bars (depicted here as gray), and ignore any blue bars (depicted here as black) in the memory array, and they had to indicate whether one of the two red bars had a different orientation in the test array

The change-detection task we used was comparable to the tasks used in Ophir et al. (2009) and Uncapher et al. (2016; Exp. 1). In Ophir et al., participants were asked to memorize the orientation of two, four, six, or eight target objects that could be shown with zero, two, or four distractor objects, and they were subsequently asked to detect the change in orientation of the targets (by 45°), which occurred on 50% of the trials. Participants completed 200 trials in total. In Uncapher et al., participants were asked to memorize the orientation of two target objects that could be shown with zero, two, four, or six distractor objects, and they were subsequently asked to detect the change of orientation of the targets that occurred on 50% of the trials. Exactly like in our study, participants in Uncapher et al. completed 200 trials in total. In our change-detection task, participants were asked to memorize two target objects (red rectangles) that were shown together with 0, 2, 4, or 6 distractor objects (blue rectangles) and to detect whether or not one of the targets changed its orientation in a subsequent display (see Fig. 1). The targets and distractors were randomly distributed in a 4 × 4 grid of an 800 × 800-pixel display, and each could have an orientation of 0, 45, 90, or 135° relative to a vertical axis. For data collected by students using their own laptop or computer, the size of display was not adjusted depending on the display resolution, meaning that the size of the display on the monitor could vary for data collected by students.

Figure 1 shows the order of the stimuli in one trial. Each trial began with a presentation of a fixation cross. Participants started the trial sequence by pressing the spacebar. The fixation cross then remained in view for another 400 ms before the memory array display was presented for 100 ms. The memory array consisted of two target objects (red bars, illustrated in grey in Fig. 1) and 0, 2, 4, or 6 distractor objects (blue bars, illustrated in black in Fig. 1).

During the memory-array presentation, participants had to memorize the orientations of the targets while ignoring any distractors. Following the memory array, there was a blank retention interval of 900 ms before the test array was presented for 2,000 ms. During the presentation of the test array, participants had to indicate whether the orientation of one of the targets had changed by pressing the left (change) or right (no change) arrow key on the keyboard. On 50% of the trials, one of the targets changed its orientation by either 45° or 90° in a clockwise or counterclockwise direction. In the remaining 50% of the trials, no change occurred. The different trial types (change or no change, with 0, 2, 4, or 6 distractors) were randomly intermixed in the experiment. In total, the experiment consisted of 200 trials with 25 repetitions of each combination of change (present vs. absent) and distractor set size. The experiment took 15–25 min to be completed.

#### Thought probes

Typically, the presence of mind-wandering during a task is gauged with experience-sampling methods. In these methods, participants are asked to indicate whether mind-wandering has occurred at a particular moment (Smallwood & Schooler, 2015).

In the current study, we deployed two types of experience-sampling probes after each block of 16 trials, thus yielding a total of 12 measurements of mind-wandering during the change-detection task.^1^ The first type of probe asked participants to rate whether their focus of attention in the preceeding block was on- versus off-task on a 7-point scale – on-task, closer to 7 or off-task, closer to 1 – and the second type of probe gauged the participants’ ability to notice the fluctuations of their focus of attention (i.e., their meta-awareness; see Christoff, Gordon, Smallwood, Smith, & Schooler, 2009; Schooler et al., 2011) also on a 7-point scale – aware, closer to 7 and unaware, closer to 1. Except for the last block of eight trials, each block included 16 trials for every combination of change (present vs. absent) and distractor set size. The last block of eight trials included two trials for each of these combinations.

### Data analysis

Following Ophir et al. (2009) and Uncapher et al. (2016), performance on the change-detection task was computed in terms of *Cowan’s K* (see Cowan, 2000)*,* with *K = S* * (*H* - *F*), with *K* denoting the number of targets retained in memory, *S* denoting the number of targets shown, and *H* and *F* denoting the hit and false-alarm rates, respectively.

We constructed linear mixed-effects models (LMEs) to test the external and internal distraction hypotheses. In addition to estimating the variabilities in the dataset related to the effects of interest (e.g., distractor set size, MMS), LMEs also allow for estimating variabilities that should be generalized over a larger population (called random effects, e.g., different participants, different stimuli used in the experiment; Baayen, Davidson, & Bates, 2008). To ensure that our findings were not affected by potential confounding variables, we performed the hypothesis testing for both the external and the internal distraction hypotheses while controlling for Age, Sex, and Testing location variables as additional fixed-effects.

The external distraction hypothesis would predict that HMMs are more affected by the distractors than the LMMs, thus resulting in an interaction of media multitasking and the effect of distractor set size. We tested this hypothesis in a model with MMS and distractor set size as fixed effects, subject as a random intercept effect, and *K* as the outcome variable. Specifically, we tested whether the addition of an interaction effect between MMS and distractor set size improved the model compared to the model without the interaction, reflecting the idea that HMMs are more affected by the number of distractors than LMMs. In examining the internal distraction hypothesis, we first tested whether the addition of MMS as a fixed effect improved the model, as would be expected if participants with a higher MMS performed worse overall. If MMS predicted *K,* we further planned to perform a mediation analysis by adding the occurrence of task-unrelated thought as a fixed effect. If the internal distraction hypothesis was correct – that is, if any deficit in performance for HMMs could be explained by the increase of task-unrelated thoughts – we should see (1) a positive correlation between MMS and the occurrence of task-unrelated thoughts and (2) an absence of predictiveness of MMS for *K* once we control for task-unrelated thoughts.

To evaluate the significance of our effects of interest, we assessed whether the addition of the relevant fixed effects improved the fit of the model by means of model comparison. Specifically, we used the *p-*values of the goodness-of-fit χ^2^ test of the relevant model comparison as the index of whether our model provided support for the external or internal distraction hypothesis. The χ^2^ goodness-of-fit test evaluates whether the model has been improved, with significant χ^2^ indicating that a larger amount of variance can be explained by adding the relevant fixed effects.

To examine whether the data provided evidence for the null hypothesis of no association between media multitasking and internal or external distraction, we used Bayes factors. Unlike the traditional approach of null hypothesis significance testing (NHST), in which only the likelihood of the data under the null hypothesis can be calculated (Wagenmakers, 2007), a Bayes factor analysis allows one to assess the evidence in favor of both the null hypothesis *H*_*0*_ and alternative hypothesis *H*_*1,*_ given a certain distribution for the prior probability of these hypotheses. Specifically, a *BF*_*10*_ expresses the ratio of the likelihood of the data under *H*_*1*_ over *H*_*0*_, while *BF*_*01*_ expresses the ratio of the likelihood of the data under *H*_*0*_ over *H*_*1*_. Thus, the Bayes factor expresses the extent to which belief in *H*_*0*_ versus *H*_*1*_ should change in view of the data.

Lastly, since Ophir et al. (2009) performed their analysis only on the extreme groups of multitaskers (i.e., HMMs and LMMs), we performed an additional analysis using a similar technique, namely categorizing the media multitaskers into HMMs and LMMs and then constructing a repeated-measures ANOVA with *K* as the outcome variable, Distractor Set Size as a within-group factor, and Group (HMM vs. LMM) as a between-group factor (see also Uncapher et al., 2016). This analysis was preregistered on the Open Science Framework: https://osf.io/nkdw5/. Further elaborations on the method used for classifying HMMs and LMMs can be found in the Supplementary Materials (p. S3).

All analyses were conducted using R 3.4.1. in RStudio 1.0.153. The linear mixed-effect models were constructed using the lme4 package (Bates, Mächler, Bolker, & Walker, 2015) and the Bayes factors were calculated using the BayesFactor package (Morey, Rouder, & Jamil, 2015). Plots were rendered using the ggplot2 package (Wickham, 2010). All significant and non-significant results were reported.

## Results

To test the presence of associations between MMS, distractor set size, and performance, we constructed and compared several LMMs. Table 1 shows the constructed models and effects tested in each model. Note that all models have Subject as a random factor and have controlled for Age, Sex, and Testing location by including those as additional fixed effects.

**Table 1:** Fixed effects tested in different linear mixed-effects models

Model	Fixed effects
m0	-
m1	Distractor set size
m2	MMS
m3	Distractor set size + MMS
m4	Distractor set size + MMS + (Distractor set size× MMS)
m5*	Distractor set size + MMS + (Distractor set size× MMS) + Focus of attention
m6*	Distractor set size + MMS + (Distractor set size× MMS) + Focus of attention + (MMS × Focus of attention)
m7*	Distractor set size + MMS + (Distractor set size× MMS) + Focus of attention + (Distractor set size× MMS × Focus of attention)

× indicates an interaction

*Models m5–m7 were part of an exploratory analysis, for which we report the results in the Supplementary Materials

### External distraction

To test the external distraction hypothesis, we started with analyzing whether performance was modulated by distractor set size. A comparison between models m1 and m0 showed that adding Distractor set size as a fixed effect significantly improved the model, *χ*^*2*^(3) = 31.12, *p* < .001, *BF*_*10*_ = 430.27. Specifically, for each distractor condition, *K* was significantly lower than for the no-distractor condition, *t’*s < -4.49, indicating that participants performed worse in the presence of distractors.

**Fig. 2:**
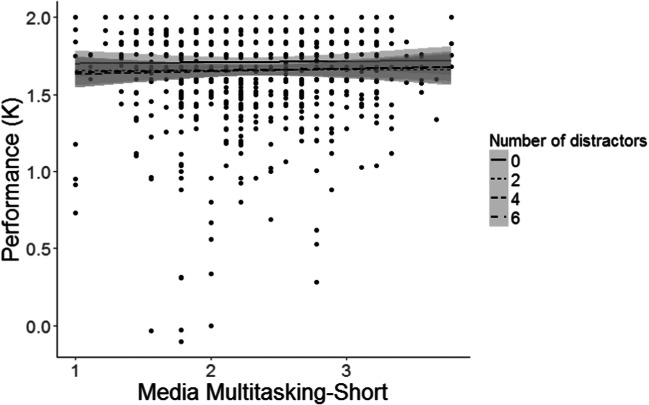
A scatterplot showing the association between MMS and the average K with different fits for distractors set size equals zero, two, four, and six. Each dot represents performance of one participant in one condition. The shaded area represents the 95% confidence interval of the mean

Subsequently, we compared the model that included the interaction between MMS and Distractor set size with the model that did not include this interaction, namely models m4 and m3, respectively. As Fig. 2 suggests, adding the MMS × Distractor set size interaction did not significantly improve the model*, χ*^*2*^(3) = 1.19, *p* = .754. In fact, the model without this interaction proved to provide a much better fit than the model with the interaction, *BF*_*01*_ = 3698.41.

Taking the same approach as Ophir et al. (2009), we ran an additional analysis using a repeated-measures ANOVA with distractor set size as a within-subject factor, media-multitasking group (HMM; N=35 vs. LMM; N=41) as a between-subject factor, and *K* as the outcome variable. Consistent with our linear mixed-effects models analyses, this analysis also showed an effect of Distractor set size, *F*(3, 222) = 3.23, *p* = .023, partial η^2^ = .042, but no significant Media multitasking × Distractor set size interaction, *F*(3, 222) = .416, *p* = .741, partial η^2^ = .005, and the Bayes factor indicated that there was solid evidence for the absence of this interaction, *BF*_*01*_ = 24.39.

### Internal distraction

#### Effects of MMS

To examine the internal distraction hypothesis, we first tested whether the addition of MMS significantly improved the model with Distractor set size only (m1). Thus, we compared models m3 and m1. This comparison showed that adding MMS as a fixed effect did not significantly improve the model, *χ*^*2*^(1) = 2.24, *p* = .121. Again, there was more support for the model without an effect of MMS than for the model that included this effect, *BF*_*01*_ = 2.70, thus providing evidence against the internal distraction hypothesis. Consistent with the outcomes of the linear mixed-effect models, an extreme-groups comparison also showed no significant difference in *K* between HMMs and LMMs*, F*(1, 74) = .61, *p* = .440, partial η^2^ = .008, *BF*_*01*_ = 2.06.

#### Mind-wandering

Our results showed no correlation between media multitasking and overall performance in the change-detection task. Thus, it was not possible to perform the mediation analysis to examine what portion of the amount of variance in the association between media multitasking-overall performance correlation could be attributed to the presence of task-unrelated thought, since there was no variance to explain. Nevertheless, we did conduct an additional exploratory analysis on the relationship between mind-wandering, media multitasking, and performance on the change detection task.

To check whether participants meaningfully interpreted the thought probes, we assessed the extent to which mind-wandering was correlated with task performance. Specifically, we first examined whether, as in previous studies, a low focus of attention was associated with more errors and faster response times (see Smallwood & Schooler, 2006). This was indeed the case, as responses were less accurate and slower in the blocks in which participants reported a lower focus of attention (see the Supplementary Materials for the associated statistics; p. S4). This confirms that participants meaningfully interpreted the thought probes.

**Fig. 3:**
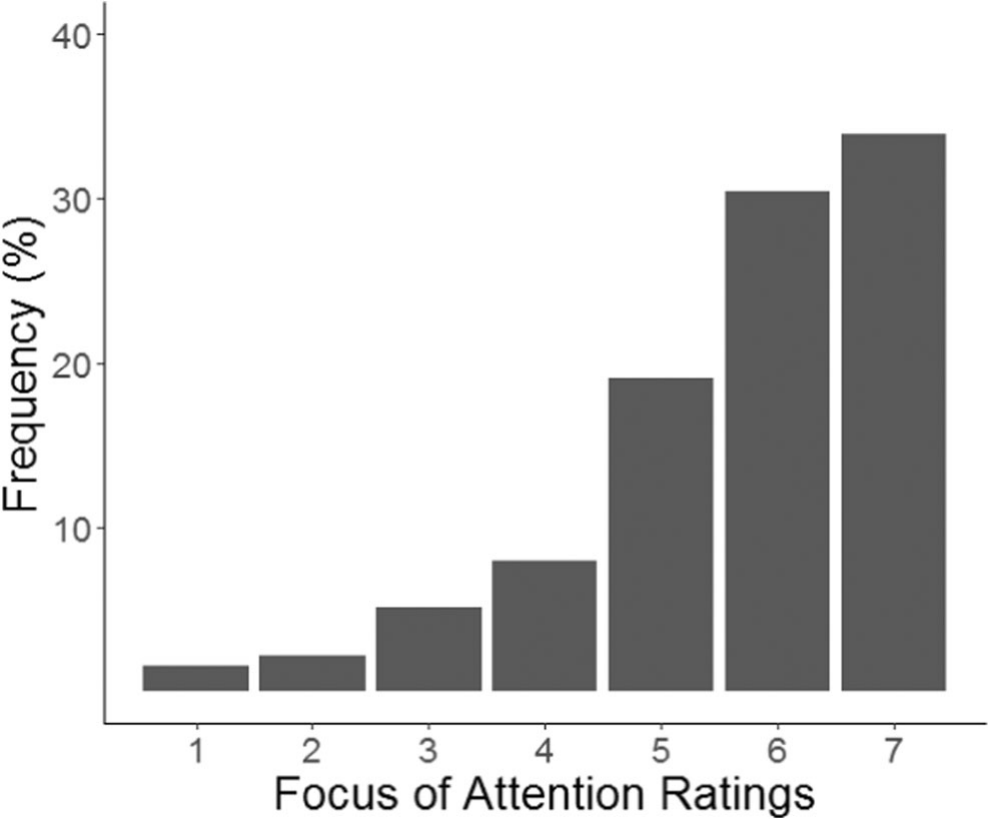
Frequency (%) of responses to focus of attention probes. Higher ratings indicate higher focus/absence of mind-wandering and lower ratings indicate lower focus/presence of mind-wandering

Next, we examined the degree of mind-wandering. As can be seen in Fig. 3, participants were focused on the task in most of the trial blocks: Across 12 blocks, participants reported being off-task (defined as reporting a rating below 4) on 8.63% of the blocks and on-task (reporting a rating above 4) on 83.42% of the blocks. Since the frequency of off-task blocks was low and since the responses for the first (on-task vs. off-task) and second (aware vs. unaware) probes were highly correlated, *r*(259) *=* .71, *p* < .001, we did not perform any further analyses for the awareness probes.

Next, we examined the correlation between MMS and Focus of attention by constructing a linear-mixed-effects model with Focus of attention as the outcome variable, MMS as fixed effect, Subject as a random intercept, while controlling for Age, Sex, and Testing location as additional fixed effects. The results showed that adding MMS as a fixed effect did not significantly improve the model, *χ*^*2*^(1) = 1.41, *p = .*236, *BF*_*01*_ = 2.43, indicating that participants with higher MMS did not mind-wander more frequently during the experiment.

#### Auxiliary exploratory analyses

Lastly, we also conducted a number of auxiliary analyses to examine the influence of a number of methodological details that differed between our change-detection task and the tasks used in previous studies. Specifically, our study differed from previous studies in that our change-detection task was self-paced (i.e., participants initiated each trial by pressing the spacebar), and in that it included a varied, as opposed to a fixed, degree of rotation for the target on change trials. In addition, our study differed from previous studies in that we used a sample of participants that not only included university students but also members from the more general population who were tested by students (see the Supplementary Materials for details on the demographics of these participants). The exploratory and auxiliary analyses showed that none of these factors appeared to be of influence on the relation between media multitasking and task performance (see Supplementary Materials, p. S8–S9). Specifically, we found that the results did not depend on how much time participants took to initiate a trial. In addition, they showed that the results did not differ depending on whether the target changed by 45° or 90° on change trials, and they also made clear that the results obtained in the main analyses were consistent when considering different subsets of participants separately. Taken together, these exploratory analyses corroborate the findings we obtained in our main analysis.

## General discussion

Previous studies have reported mixed findings on the association between media multitasking and performance in laboratory tests of attention, working memory, and cognitive control. Specifically, some studies suggest that HMMs are more vulnerable to distractors present in the immediate environment (the external distraction hypothesis), whereas others suggest that HMMs perform worse overall, regardless of the presence of distractors, due to the increased vulnerability to internal distraction (the internal distraction hypothesis), and yet others found no evidence for these associations. In the current study, we tested these possibilities in a large-scale experiment in which we collected data both from university students and members of the general population. In addition, we included thought probes to enable us to determine whether any reduction in performance could be ascribed to an increase in task-unrelated thought. In examining the evidence for the internal and external distraction hypotheses, we employed different analysis methods; we performed a repeated-measures ANOVA for an extreme-groups comparison as well as a linear-regression analysis across all participants, and we complemented the use of null-hypothesis significance tests with Bayes-factor analyses.

Overall, we found consistent evidence that media multitasking was not associated with task performance in a change-detection task. Specifically, while we did find that participants performed worse as distractor set size increased, we did not find that participants with higher media multitasking scores were more strongly affected by the presence of distractors. Thus, in this regard, our findings failed to corroborate Ophir et al.’s (2009) findings that HMMs perform worse as distractor set size increases and they instead corroborated the results of other studies that also did not report this interaction (see Wiradhany & Nieuwenstein, 2017 for a review). We also found that media multitasking was not associated with worse overall performance in the change-detection task. This result appears to be at odds with the findings of Uncapher et al. (2016; see also Wiradhany & Nieuwenstein, 2017, Exp. 1), whereas it corroborates earlier findings showing no association between media multitasking and overall performance in a change-detection task (Cardoso-Leite et al., 2015; Gorman & Green, 2016; Wiradhany & Nieuwenstein, 2017, Exp. 2). Lastly, we found no association between media multitasking and mind-wandering, thereby corroborating an earlier study that also failed to observe this association (Ralph et al., 2015), and thereby providing additional evidence counter to that of two previous studies that did suggest an association between media multitasking and mind-wandering (Loh et al., 2016; Ralph et al., 2013).

At present, our findings add to the mixed findings with regard to the association between media multitasking and change-detection performance in particular. Specifically, of the seven studies reported in the literature, one reported an association between media multitasking and increased distractibility (Ophir et al., 2009), three reported an association between media multitasking and worse overall performance (Uncapher et al., 2016, Exps 1 & 2; Wiradhany & Nieuwenstein, 2017, Exp. 1), and four showed neither increased distractibility nor overall worse performance in heavy media multitaskers (Cardoso-Leite et al., 2015; Gorman & Green, 2016; Wiradhany & Nieuwenstein, 2017, Exp. 2). To account for these mixed findings, two points are worth discussing, namely the fact that the current study differed from previous studies in terms of having considerably greater statistical power than all previous studies, and, secondly, that the current study differed from previous studies in using a short as opposed to a long questionnaire to measure media multitasking.

As a result of including a large number of participants, our study had considerably greater statistical power than previous studies (Cohen, 1992). The issue of statistical power is an important one since previous studies have indicated that the association between questionnaire measures of media multitasking and lab-based measures of distractibility is probably very weak (Wiradhany & Nieuwenstein, 2017). Thus, a larger sample size and greater statistical power would be needed to be able to reliably detect these effects (Button et al., 2013). As an indication of the reliability of our findings, we can estimate the statistical power we had, based on the most reliable estimates of effect sizes for the MMI – change detection performance link, namely from Experiments 1 and 2 from Uncapher et al., 2016 (N=139). Here, since Uncapher et al. reported correlations of .19 and .16 for the associations between MMI and *Cowan’s K* in their first and second experiments, respectively, our current sample size of 261 participants would provide a statistical power of .81 and .74 to detect those effects. Together, the facts that the current study had acceptable statistical power (~80%) to detect the effect shown in the previous study with the largest N and that we found null results that indicate that either the true association between media multitasking and change-detection task performance is very small (and thus, a study with an even larger sample is needed to detect the effect) or that there is no association.

While one interpretation of the current findings would be that the association between MMI and change-detection performance is null or close to zero, it is also possible that we did not find the association due to some differences between our study design and those of earlier studies that did show this association. An important alternative explanation for why the findings of the current large-scale study did not show the associations found in some previous studies is that we used the short MMS, as opposed to the long MUQ questionnaire (Ophir et al., 2009), which was used in all previous studies on change-detection performance. Since the short MMS includes only nine of the 144 media pairs that are included in the long MUQ and since the short MMS was validated in a sample of 11- to 15-year-olds, it could be that the short MMS does not probe those behaviors that might have driven the association between distractibility and media multitasking found in some previous studies. While this indeed constitutes a logically possible account that awaits an empirical test, there are several reasons why this account is unlikely to provide a satisfactory explanation for why our findings differed from those of some previous studies. To start, while the MMS is indeed short, it is important to note that Baumgartner et al. (2017b) found that the nine media pairs included in the MMS produced a score that was highly correlated (*r* = .82) with a score that was derived from a larger questionnaire that included a total of 72 media pairs from Baumgartner et al. (2014) that included a total of 72 media pairs, which also featured in an even longer questionnaire used by Ophir et al. (2009). With regard to fact that MMS was validated in a large sample of adolescents, a reanalysis of multiple MUQ datasets in a recent study has shown that both adults and teenagers have comparable media multitasking habit patterns (Wiradhany & Baumgartner, 2019). Together, it is unlikely that our use of MMS would lead to markedly different results than the MUQ. Additionally, it is important to note that the studies that did use the original MUQ have also produced highly variable results, with the majority showing null effects and only some showing evidence for a statistically significant association (see Wiradhany & Nieuwenstein, 2017, for a meta-analysis). Therefore, it is more likely that the true association between media multitasking and distractibility is null or very small than it is with the possibility that the original MUQ captures variance in some types of media-multitasking behaviors that indeed relates to performance on laboratory tests of distractibility. As stated, however, the currently available evidence does not include any empirical test of whether different types of media-multitasking behaviors might relate to distractibility to different degrees, and, therefore, a clear conclusion on this issue will have to await further research.

Taken together, the data presented in this study provide evidence against both the external and internal distraction hypotheses. Against the external distraction hypothesis, our findings corroborated the results of our recent meta-analysis, which suggested that previous evidence for the external distraction hypothesis was weak and driven primarily by studies using relatively small sample sizes (Wiradhany & Nieuwenstein, 2017). By implication, our findings also argue against the breadth-biased (Lin, 2009) and reduced top-down control (Cain & Mitroff, 2011) accounts, which would both predict that participants with higher MMS scores would be more strongly affected by distractor set size than those with lower MMS scores, due to their tendency to absorb as much information as possible or due to a reduced ability to exert top-down control.

Against the internal distraction hypothesis, our finding that media multitasking is not associated with worse overall performance corroborated other studies in the literature that found no association between media multitasking and performance a change-detection task (Cardoso-Leite et al., 2015; Gorman & Green, 2016; Wiradhany & Nieuwenstein, 2017, Exp. 2), an N-back task (Baumgartner et al., 2014; Edwards & Shin, 2017; Wiradhany & Nieuwenstein, 2017), sustained-attention tasks (Ralph et al., 2015), a task-switching paradigm (Alzahabi et al., 2017; Baumgartner et al., 2014; Minear et al., 2013), an Eriksen flanker task (Baumgartner et al., 2014; Murphy et al., 2017), and a Go/noGo task (Murphy et al., 2017; Ophir et al., 2009). In addition, our finding that media multitasking is not associated with increase of mind-wandering corroborated other studies that also found no evidence for a media multitasking-mind-wandering association (Ralph et al., 2015). Together, this set of findings oppose what has been proposed in a recent review (Uncapher & Wagner, 2018). This review suggests that there was converging evidence in the literature that media multitasking is associated with worse task performance, especially those related to retaining information in memory, and that this might be due to the higher number of attentional lapses experienced by frequent media multitaskers. Critically, the considered evidence in this review was based on numerical as opposed to statistical differences in task performance between HMMs and LMMs. Indeed, in cases in which only statistical evidence were considered, there has been a weak support for the attentional lapses account, and furthermore, our current findings provided direct evidence against the notion that (1) HMMs performed worse than LMMs and (2) HMMs experienced more frequent attentional lapses.

### Limitations and future directions

While we showed no evidence for the external and internal distraction hypotheses, several cautionary notes should be kept in mind. To start, some of our data were collected outside the laboratory, and while we instructed our students to follow a strict procedure, some data collection settings, such as the screen size of the laptops and other possible differences across experimenters, remained uncontrolled and could thus affect our results. At the same time, collecting data outside the laboratory allowed us to collect a large amount of data more efficiently, and several additional tests (p. S1-S2 in the Supplementary Materials) suggested that the data from and outside the lab were of a similar quality.

Our study design also has several differences compared with those in the current literature that may limit the comparability of our results with previous ones and may contribute to our null findings. In addition to our use of the short MMS, our change-detection task was also not designed *a priori* to detect effects of mind-wandering. As such, in our case, the participants on average reported low degrees of mind-wandering during the experiment. It could be the case that due to the combination of low incidence of mind-wandering and a possible small true effect of MMI on mind-wandering, we might not have had adequate statistical power to detect the association between MMI and mindwandering.

Since we assessed media multitasking using only a small subset of all possible media-multitasking behaviors, an important question for future studies will be to examine whether associations between cognition and media multitasking do exist for other types of media-multitasking behaviors. Furthermore, in conducting these studies it is also important to consider that people tend to underestimate their frequency of switching between media streams (Brasel & Gips, 2011) and that they tend to overestimate the time they spend using media (Deng, Meng, Kononova, & David, 2018). Another recommendation for future studies would therefore be to combine the use of self-report measures with the use of more objective methods such as diaries (Voorveld & Goot, 2013; Wang & Tchernev, 2012), video recordings of behavior (Rigby, Brumby, Gould, & Cox, 2017), and, especially, automatic tracking on a participant’s devices (Wang & Tchernev, 2012; Yeykelis, Cummings, & Reeves, 2014). Additionally, future studies might be interested in examining the association between media multitasking and task performance using a more complex working-memory task, as studies using this type of performance measure have shown more robust associations (Cain et al., 2016; Ralph & Smilek, 2016; Sanbonmatsu et al., 2013).

By combining these objective measures of media multitasking with self-report measures, by considering whether different types of media-multitasking behaviors produce different results, and by examining the type of task which differentiate heavy from light media multitaskers, we believe that future studies could make an important contribution towards uncovering the existence of any associations between habitual media multitasking and laboratory measures of information processing and distractibility in this exciting and increasingly important scientific field.

### Conclusion

To conclude, the current large-scale study showed that media multitasking, as assessed using the nine media pairs of the MMS (Baumgartner et al., 2017b), is associated with neither increased vulnerability to external distraction nor reduced performance due to the occurrence of internal distraction.

## Electronic supplementary material

ESM 1 (DOCX 58 kb)

## Footnotes

1 Note that since the number of trials is 200, the last block only has a set of eight trials. We did not include thought probes after this last block.
