# Supplementary material for: Media multitasking, mind-wandering, and distractibility: A large-scale study
Source: Atten Percept Psychophys. 2019 Aug 7;82(3):1112–24. doi: 10.3758/s13414-019-01842-0 (PMC7303060; doi:10.3758/s13414-019-01842-0)
Supplement: Supplementary file 1 — (DOCX 58 kb) [file 13414_2019_1842_MOESM1_ESM.docx]

Supplementary Materials for Media multitasking, mind-wandering, and distractibility: A large-scale study

**Data Collected in the Lab or by Students**

To examine whether the two samples of participants in our study differed, we compared the demographics, media-multitasking indices, and performance of participants for whom the data was collected in the lab (N = 107) or by students using their own computers and laptops (N = 154; see Table S1). As can be seen in Table 1, all measures showed a statistically significant difference between the data collected in the lab and the data collected by students. Specifically, the percentage of female participants was higher for the data collected in the lab, and the participants in the lab were significantly younger, with more variance of age in the data collected by students outside of the lab. In addition, the participants tested in the lab had a higher media-multitasking score, a higher frequency of mind-wandering (less attentional focus on task), and worse performance on the change detection task. Importantly, however, our analyses of the relationship between media multitasking, mind-wandering, and change detection showed no significant effects of the setting of data collection. Specifically, a linear mixed effects model with Testing location, MMS, and Number of distractors as fixed factors, Subject as a random factor, and *K* as the outcome variable showed that testing location did not interact with MMS, *χ^2^*(2) = 5.32, *p* = .070, *BF_01_* = 3.23, *t = .*77 or with MMS × Number of distractors, *χ^2^*(8) = 8.59, *p* = .378, *BF_01_* = 48.68, all *t’*s *< .*59, thus indicating that the main findings of interest were consistent across these two subsets of data.

| Measure | Lab (Mean and SD) | Own Computer (Mean and SD) | Test Statistic | *p*-value | Levene’s Test of Equality of Variance | *p-*value |
| --- | --- | --- | --- | --- | --- | --- |
| Age | 20.94 (2.65)* | 28.17 (13.36)** | *t*(168.82) = 6.47 | .001 | *F*(1, 249) = 27.48 | < .001 |
| % Female | 72% | 53% | *Wilcoxon W* = 18632 | .002 | *Fligner-Killeen more χ*^2^(1) = 9.24 | .002 |
| Media-multitasking-short | 1.42 (.51) | 1.27 (.55) | *t*(259) = 2.33 | .021 | *F*(1, 249) = .10 | .308 |
| Focus of attention | 4.53 (.85) | 4.79 (1.00) | *t*(259) = 2.16 | .032 | *F*(1, 249) = 2.27 | .133 |
| Awareness of attention | 4.52 (.93) | 4.69 (1.09) | *t*(259) = 1.35 | .178 | *F*(1, 249) = 1.68 | .196 |
| Cowan’s K | 1.63 (.23) | 1.70 (.25) | *t*(259) = 2.16 | .032 | *F*(1, 249) = .10 | .706 |

**Table S1.** Results for data collected in the lab vs. by students using their own computers and laptops. * N = 99, ** N = 152.

To further show that the main findings reported in the main text are consistent across the different testing locations, we performed the main analysis again, but this time separately for the two testing locations. In other words, we fitted linear mixed effects models with K as the outcome variable, MMS, Number of distractors, Focus of attention, MMS x Number of distractors interaction, and Focus of attention as fixed-effects, and Subject as random intercept twice, first for the subset of data collected in the lab and second for the subset of data collected outside the lab. For the data collected in the lab, we found an effect of Number of distractors, χ^2^ = 12.18, *p =* .006, but no effect of MMS, χ^2^ = .88, *p =* .349, and no MMS x Number of distractors interaction, χ^2^ = .39, *p =* .942. Adding Focus of attention significantly improved the model, χ^2^ = 93.43, *p <* .001. For the data collected outside the lab, we found an effect of Number of distractors, χ^2^ = 17.97, *p <* .001, but no effect of MMS, χ^2^ = .014, *p =* .907, and no MMS x Number of distractors interaction, χ^2^ = .91, *p =* .822. Adding Focus of attention significantly improved the model, χ^2^ = 71.44, *p <* .001. These effects replicate the main analysis on p. 19-22 of the manuscript. Together, these results further indicate that our key findings were consistent across different testing locations, both when the location was included as a factor in the analysis and when the two settings were considered as separate experiments. At the same time, we witnessed that participant’s Age and Sex varied considerably across the two datasets. Participant’s Age, in particular correlated positively with Focus of attention, *r*(249) = .23, *p* < .001. For the remaining analyses and for the main analysis reported in the manuscript which relate to Focus of attention, we controlled for participant’s Age, Sex, and Testing location as covariates.

**Extreme-group Comparisons: Identifying Heavy and Light Media Multitaskers**

For the extreme-groups comparison, which we performed to facilitate a more direct comparison with the results of Ophir, Nass, and Wagner (2009) and Uncapher, Thieu, and Wagner (2016), we first calculated the Media Multitasking Index (MMI; Ophir et al., 2009), using equation 1 below:

$$MMI =\sum_{i=1}^{j} \frac{m_{i}\times h_{i}}{h_{j}}$$

In this equation, *m_i_* is the sum of the media multitasking frequency scores using primary medium *i, j* is the total number of media evaluated, *h_i_* is the number of hours spent consuming primary medium *i*, and *h_j_* is the sum of hours spent consuming all media. The distribution of the MMI had a mean of 1.35 and an SD of .54. The scores were normally distributed, Shapiro-Wilk’s *W* = .99, *p =* .70 and they were highly correlated with the MMS, *r* = .96, *p <* 2.2e -16. Following Ophir et al. (2009), participants whose MMI lay more than one SD above the mean were categorized as Heavy Media Multitaskers (HMMs) and participants whose MMI lay more than one SD below the mean were categorized as Light Media Multitaskers (LMMs). Using this categorization, we identified 35 HMMs (*M* = 2.35, *SD* = .26) and 41 LMMs (*M* = .54, *SD* = .24).

If we used the MMS instead of the MMI as a media multitasking index, we had 46 HMMs (*M* = 3.24, *SD* = .25) and 40 LMMs (*M* = 1.55, *SD* = .23) using a similar categorization process as above. In that case, the analysis also revealed an effect of Number of distractors, *F*(3, 252) = 4.9, *p =* .002, partial η^2^ = .056, *BF*_10_ = 8.75, but no main effect of Group, *F*(1, 84) = .021, *p =* .885, partial η^2^ <.001, *BF*_01_ = 3.05, and no Number of distractors × Group interaction, *F*(3, 252) = .74, *p =* .529, partial η^2^ = .008, *BF*_01_ = 16.13.

**Supplementary Analyses for Task-unrelated Thought**

**Accuracy and Response Times**

To verify whether the participants responded seriously to the thought probes, we examined whether performance differed according to the self-reported focus of attention in the thought probes. To this end, we constructed linear mixed-models with Subject as a random intercept, Focus of attention as a fixed-effect, and Accuracy and Response times for correct trials as outcome variables, and we controlled Sex, Age, and Testing location as additional fixed-effects. Overall, the results showed that participants were less accurate in blocks in which they reported low Focus of attention, *χ^2^*(1) = 109.74, *p < .*001, *BF_10_* = 3.47e+22, see Supplementary Figure 1, but they were not slower or faster *χ^2^*(1) = 1.15, *p = .*250, *BF_01_* = 8.98.


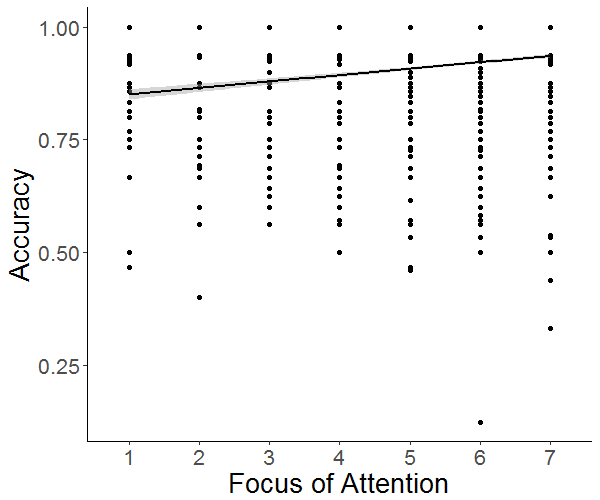


**Figure S1.** A scatterplot showing the association between Focus of Attention and Accuracy of trials following the presentation of the thought probes. Each dot represents performance of one participant in one condition. The shaded area represents 95% CI of the mean.

If we only consider the trials prior to participants responding to the probes, namely the last trial of each block, participants were more likely to respond incorrectly in trials in which they reported a lower focus of attention, *χ^2^*(1) = 10.065, *p < .*001, *BF_10_* = 29.29 (see Figure 1) and they had somewhat slower responses as well, *χ^2^*(1) = 10.67, *p = .*001, *BF_10_* = 17.74. Together, this suggests that participants responded meaningfully to the thought probe.

In addition to thought probes, another way that researchers have sought to probe distraction is to look at how performance changes over the course of the task. Specifically, focus of attention might wane as a function of time on task and this might reduce task performance. To evaluate this possibility, we fitted a linear mixed effects model with K as the outcome variable, Focus of attention and Block (as a proxy of time on task) as fixed-effects, and Subject as a random intercept, and we controlled Sex, Age, and Testing location as additional fixed-effects. We found that adding Block as a fixed-effect explained more variance, χ2 = 68.94, *p* < .001. Specifically, participants performed better in later blocks, *t*=8.31. There was a Focus of attention x Block interaction, χ^2^ = 9.52, *p =* .002; low Focus of attention was associated with lower K, and the magnitude of this association was larger in the earlier compared to later blocks. However, the Bayes Factor indicated a strong support for the null model, *BF_01_* = 6.01e+7. The effect of Block on K did not vary as the function of MMS. The model with a Block x MMS interaction did not explain significantly more variance than the model without, χ^2^ = 6.22, *p =* .859, *BF_01_* = 1.14e+12. In sum, we found effects of time on task on performance, such that participants showed an improvement in task performance, and this effect did not vary as a function of media multitasking.

**Interaction with MMS**

To examine whether or not Focus of attention interacted with MMS, we first examined whether the addition of focus of attention as a fixed effect improved the model presented in the main text by comparing models m4 and m5, which differ in whether they contained Focus of attention as a fixed-effect. This turned out to be the case, *χ^2^*(1) = 133.90, *p < .*001, *BF_10_* = 1178.94. Specifically, aligned with our finding that focus of attention was associated with a positive regression slope, *t=*11.60, this confirms that a stronger focus of attention on task is associated with better performance*.*


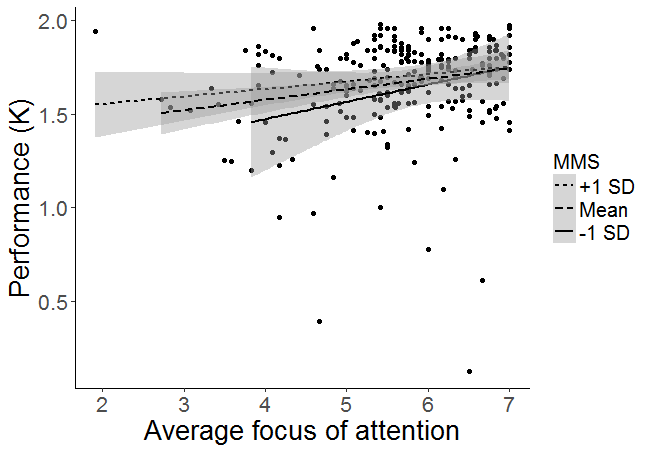


**Figure S2.** A scatter plot showing the association between the average focus of attention and K, with different fits for participants with MMS scores above 1 SD of the mean (High), participants with MMS scores between +1 SD and -1 SD of the mean (Mean), and participants with MMS scores below 1 SD of the mean (Low). Each dot represents performance of one participant at one level of focus of attention. The shaded area represents 95% CI of the mean.

We evaluated whether focus of attention interacted with MMS by comparing models m5 and m6, which differ in whether they contained MMS × Focus of attention interaction as a fixed-effect. This was the case, *χ^2^*(1) = 7.49, *p = .*006, *BF_10_* = 1.12. Specifically, the Focus of attention × MMS interaction had a negative coefficient estimate, *t* = -2.73, indicating that participants with higher MMS showed less difference in performance between being off-task and on-task compared to participants with lower MMS (see Figure 4). Lastly, we examined if there was a three-way interaction between Focus of attention, MMS and Distractor set size by comparing models m6 and m7, which differed in the presence of this three-way interaction. This was not the case, *χ^2^*(3) = 0.08, *p = .*994, and the Bayes Factor indicated that the null model was more likely *BF_01_* = 51.27.

**Effects of Self-paced Design?**

Additionally, we also checked whether our self-paced experiment design somewhat influenced the effect of Focus of attention on performance. Due to the self-paced nature of the task, participants might wait until they perceive themselves to be on-task before starting a trial. If this was the case, we should observe a positive correlation between the time it takes before participants started a trial and the Focus of attention probes on that block. We tested this in a linear regression analysis with Response time during fixation as the outcome variable, Focus of attention as the predictor, and Age, Sex, and Testing location as covariates. The results showed that Response times did not vary as a function of Focus of attention controlling for the covariates, *β = -*108.26, *t=-* 0.61, *p =* .181, *BF_01_* = 38.40, indicating that it is unlikely that the self-paced nature of the task made participants systematically wait until they were on-task before starting a new trial.

**Response Rates of Participants**

The participants included in the main analysis had a response rate of 97.61% (SD=3.88). To analyze whether response rate varied as a function of focus of attention and MMS, we binned the dataset into different blocks and included the blocks in which Focus of attention was probed. We then fitted a model with response rates as the outcome variable, Focus of attention and MMS as predictors, and Age, Sex, and Testing location as covariates. We found that response rate varied as a function of Focus of attention, *β =* 0.03, *t=* 2.69, *p =* .007, indicating that in blocks in which participants reported lower focus of attention they missed giving responses more often. It also varied as a function of MMS, *β =* 0.09, *t=* 2.56, *p =* .010, indicating that participants with higher MMS scores missed giving responses more often. Critically, however, the Bayes Factors indicated that there was only weak support for these effects, *BF_10_* = 2.37 and *BF_10_* = 1.47, respectively, thus warranting caution about their reliability.

**Stimuli Degrees of Rotation**

Our set of stimuli includes two different degrees of change of the target rotation, namely 45 and 90 degrees. Thus, participants might be more likely to detect changes of target orientation in one level compared to the other. To evaluate this possibility, we fitted a model with Correct responses as the outcome measure, the occurrence of Change with two (change, no change) or three (change 90 degrees, change 45 degrees, no change) levels as the outcome measure, and Subject as a random intercept. If performance differed in responding to 45 and 90 degrees of change, the model with three levels of change would explain more variance. We found that participants were less likely to respond correctly when Change occurred, χ^2^ = 160.75, *p <* .001, but importantly, fitting the Change predictor in three levels corresponding to the three possible orientations did not explain significantly more variance, χ^2^ = 2.56, *p =* .109, signifying that participants did not seem to perceive one level of degree of change to be more difficult or easier than the other. Additionally, MMS also did not interact with models separating the degree of Change into either two (χ^2^ = .26, *p =* .614) or three levels (χ^2^ = 1.23, *p =* .542). This indicates that Heavy or Light media multitaskers were not more sensitive to different degrees of stimulus change.

**Bibliography**

Ophir, E., Nass, C., & Wagner, A. D. (2009). Cognitive control in media multitaskers. *Proceedings of the National Academy of Sciences of the United States of America*, *106*(37), 15583–7. http://doi.org/10.1073/pnas.0903620106

Uncapher, M. R., Thieu, M. K., & Wagner, A. D. (2016). Media multitasking and memory: Differences in working memory and long-term memory. *Psychonomic Bulletin & Review*, *23*(2), 483–490. http://doi.org/10.3758/s13423-015-0907-3
